# The characteristics of video capsule endoscopy in pediatric Henoch–Schönlein purpura with gastrointestinal symptoms

**DOI:** 10.1186/s12969-020-00471-4

**Published:** 2020-10-28

**Authors:** Youhong Fang, Kerong Peng, Hong Zhao, Jie Chen

**Affiliations:** Department of gastroenterology, The Children’s Hospital, Zhejiang University School of Medicine, National Clinical Research Center for Child Health, 3333 Bin Sheng Road, Hangzhou, 310052 Zhejiang Province China

**Keywords:** Video capsule endoscopy, IgA vasculitis, Child

## Abstract

**Background:**

Henoch–Schönlein purpura (HSP) is a systemic small-vessel vasculitis also named IgA vasculitis that commonly affects the gastrointestinal tract. The video capsule endoscopy (VCE) characteristics of pediatric HSP patients are rarely reported.

**Methods:**

Patients diagnosed with HSP and analyzed by VCE examination at our hospital from February 2010 to January 2019 are enrolled. The clinical features, laboratory findings, and the characteristics of VCE findings are studied.

**Results:**

There are 30 patients enrolled in this investigation from February 2010 to January 2020. The mean age of these patients is 96.9 ± 35.8 months, and the most frequent finding of VCE is mucosal erosion, which account for 79.3% of the patients, and followed by mucosal erythema or petechia accounted for 69% of the patients. Regarding the disease location detected by endoscopy, jejunum is the most common involved part of the gastrointestinal tract in pediatric HSP patients. All the patients had the jejunum involved except in one patient the VCE did not pass through the pylorus. One third of the patients involved the descending portion of duodenum. No side effect is observed in this study.

**Conclusions:**

VCE may be an excellent adjust tool for evaluation of the gastrointestinal tract in children with abdominal symptoms without typical purpura in suspected pediatric HSP patients. VCE appears to be superior to esophagogastroduodenoscopy in detecting small intestinal lesions of HSP and has an excellent safety profile.

## Background

Henoch–Schönlein purpura (HSP) is a systemic small-vessel vasculitis that often affects children and occasionally affects adults. It is named Ig A vasculitis as well. It is more common in the Asian population than the Western population. HSP mainly involves the small vessels of skin, joints, gastrointestinal (GI) tract, and kidney. About 50% to 85% of the HSP patients have GI symptoms [1]. GI symptoms include acute abdominal pain, nausea, vomiting, hematochezia or melena, and diarrhea. However, endoscopic evaluations are not performed in all HSP patients with GI symptoms. Among the patients with GI symptoms, there is a rare portion of patients without skin manifestations, who may benefit from endoscopy evaluation. HSP most affects the descending part of the duodenum in adult patients, and the typical findings of endoscopy of HSP are erythema, petechia, erosion and ulceration [1, 2]. Although esophagogastroduodenoscopy (EGD) is useful in the diagnosis of the majority HSP patients affect GI tract, these patients may benefit from video capsule endoscopy (VCE) examination to evaluate the mucosa of small bowel.

There are several reports previously published that detailed the endoscopy findings of child and adult HSP [2]. However, there is no report focuses on the diagnosis value of VCE in pediatric HSP patients. Here we report a cohort of pediatric HSP patients who had a VCE examination. All the patients are diagnosed with HSP and had GI symptoms. We summarize the clinical and VCE findings of these patients in this article.

## Materials and methods

We enrolled 30 HSP patients who had a VCE examination at Children’s Hospital, Zhejiang University School of Medicine from February 2010 to January 2019. The clinical features, laboratory findings, and the findings of VCE were reviewed. This study was approved by the Ethics Committee in Children’s hospital, Zhejiang University School of Medicine.

All the patients had EGD examination and had biopsy at the duodenum and antrum. The indications for VCE examination were as following: 1. Patients who had suspected HSP but without typical skin palpable purpura and EGD did not reveal characteristic findings of HSP. 2. HSP patients with GI symptoms but were not completely responsive to steroids treatment or steroid-dependent. 3. Patients who had suspected HSP but needed to be differentiated with other small intestinal diseases. Patients with massive intestinal bleeding who could not tolerate VCE examination or with acute surgical indications were excluded. Small bowel radiography, magnetic resonance enterography (MRE) or computed tomography (CT) were performed to exclude a gastrointestinal stricture before the VCE examination. The device used for VCE was OMOM (Chongqing, China). The capsule was either swallowed by the patients or was delivered by EGD in patients who could not swallow the capsule.

### Statistical analysis

The continuous variables with normal distribution were presented as mean ± SD, otherwise were presented with median ± interquartile range (IQR); discontinuous variables were presented as number or percentage. The statistical analyses were conducted with SPSS 22.0 statistical software (SPSS Inc., IBM Corp., Armonk, NY, United States).

## Results

### The demographic features of the patients

Thirty patients had VCE examination for suspected HSP from February 2010 to January 2020. The demographic characteristics of these patients were showed in Table 1. All the patients had GI symptoms, including abdominal pain, vomiting, or intestinal bleeding. Half of patients had purpura at admission or during hospitalization. Forty-three percent of patients had typical purpura, and two patients were reported to have purpura but not observed by a provider. The GI symptoms and other symptoms of these patients were also shown in Table 1. The complications included hypertension, appendicitis, acute pancreatitis and acute intestinal perforation. The patient with intestinal perforation had surgery. Five patients had a history of HSP previously. One patient had a relapse of HSP one year later.

**Table 1:** The demographic characteristics and laboratory findings of HSP patients

Items	Results
Age (M)	96.9 ± 35.8
Male/Female	19/11
Median time of disease at admitted (Day)	12.5 (IQR: 6.5–20.0)
Median time perform VCE examination (Day)	24.5 (IQR:16.3–42)
Purpura, n (%)	15 (50)
Abdominal pain, n (%)	30 (100)
Vomiting, n (%)	9 (30)
Intestinal bleeding, n (%)	8 (26.7)
Arthralgia, n (%)	4 (13.3)
Hematuresis/proteinuria, n (%)	3 (10.0)
WBC (×10E9/L)	17.4 ± 7.1
Hb (g/L)	128.4 ± 11.7
PLT (×10E12/L)	372.3 ± 111.2
CRP (mg/L)	5.5 (IQR: 0.8–20.8)
ESR (mm/h, *n* = 24)	8 (IQR: 6.0–20.8)
Serum albumin (g/L, *n* = 23)	36.4 ± 6.8
Plasm D-dimers (mg/L, *n* = 22)	2.2 (IQR: 0.5–8.4)
Plasma IgA level (g/L, *n* = 19)	1.9 ± 0.8

*HSP* Henoch–Schönlein purpura; *WBC* White blood cell; *Hb* Hemoglobin; *PLT* Platelet; *CRP* C-reactive protein; *ESR* Erythrocyte sedimentation rate

### The laboratory findings of the HSP patients

The laboratory results of these HSP patients were listed in Table 1. Mean count of white blood cells and serum plasm D-dimers levels were elevated, and median CRP and ESR levels were normal.

### Images of HSP patients

Fourteen patients had MRE or CT scans of abdominal. Ten patients had bowel wall thickening alone. No other patients had scan abnormalities.

### Features of endoscopy findings

All patients had an EGD exam with biopsies performed in the gastric antrum and duodenum. Among them, 27 patients had first EGD in our hospital, three patients had EGD in other hospitals before admission, and one patient had second EGD in our hospital. No typical traits of HSP were detected by the EGD examination in 19 patients. One patient revealed duodenum ulcers by first EGD examination at the acute stage of diseases, while the second EGD examination in our hospital 5 months later was normal. The most frequent findings of EGD were mucosal ecchymosis, petechiae, erosion, and multiple ulcers. EGD revealed typical traits of HSP in the descending portion of the duodenum in nine patients. Two patients had the whole stomach involved, and one also had the lower part of the esophagus involved. Thirteen patients performed colonoscopy, and two patients detected ulcers in the terminal ileum. One patient exhibited a polyp in the colon which was felt to be an incidental finding.

**Fig. 1:**
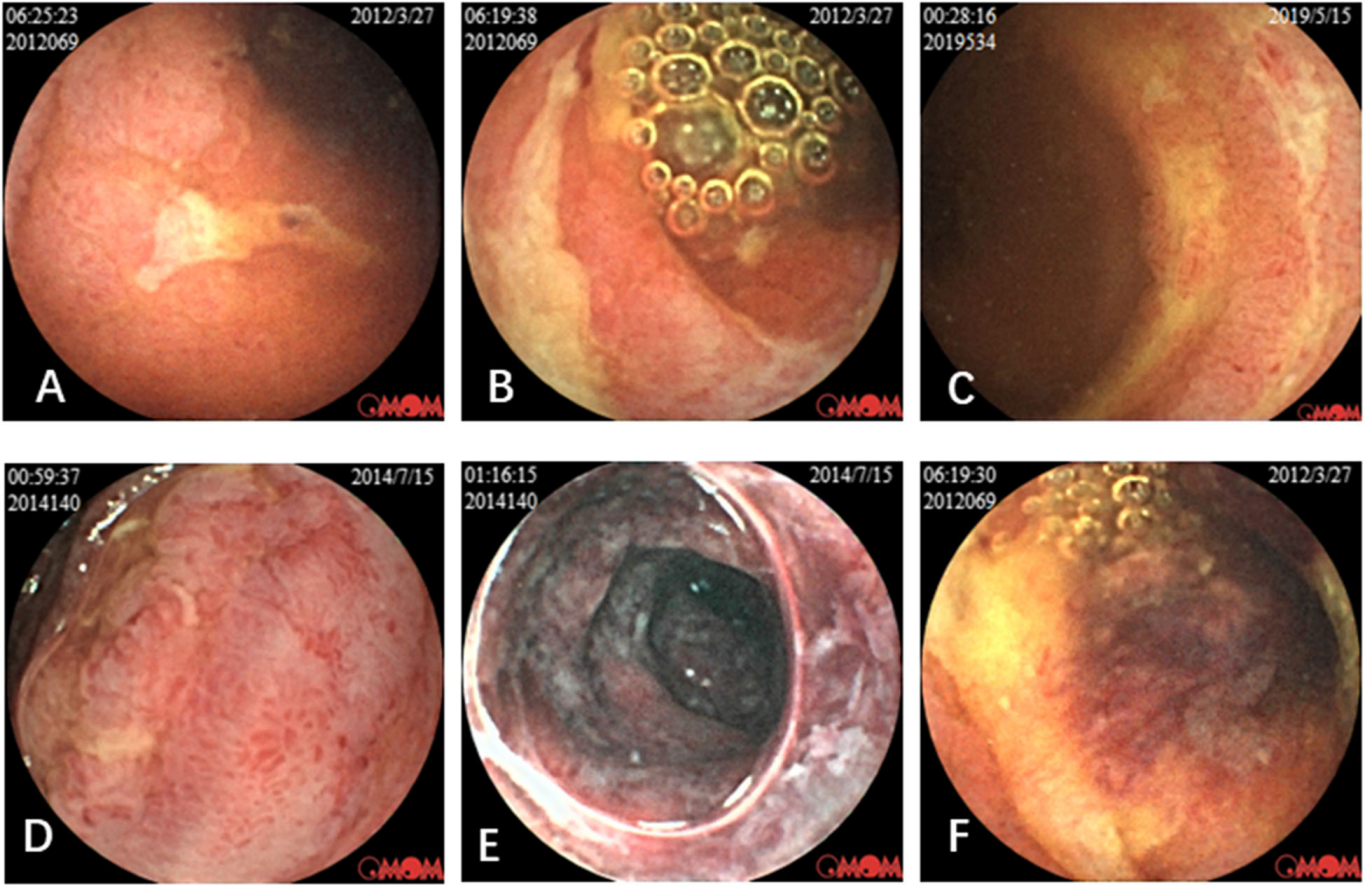
The video capsule endoscopy showed mucosal edema, congestion, erythema, petechia, diffuse erosion and multiple irregular superficial ulcers at small bowel in pediatric HSP patients

Thirty patients had a VCE examination. The Capsule did not pass through pylorus in one patient, and the others all went through the whole small bowel. The median time of the VCE examination was 21.0 days (IQR: 13.8 to 36.0) after the initial symptoms of HSP appeared. VCE detected multiple mucosal ecchymosis, erosion, and irregular superficial ulcers, which resembled the findings of EGD in 27 patients. Moreover, some patients with massive intestinal bleeding tended to have diffuse erosions and large areas of ulcers (Fig. 1). The numbers and percentage of different lesions identified by VCE were listed in Table 2. One male patient had massive intestinal bleeding, and intestinal perforation was treated with surgery and followed with oral methotrexate (MTX). He had a VCE examination to assess the recovery of intestinal lesion eight months after the onset of disease, and the VCE only detected mild mucosal congestion in the jejunum. The disease location of patients detected by endoscopy was shown in Table 3. The most frequently involved disease location in this cohort was jejunum, which account for 96.7% of the patients, and followed by the descending part of the duodenum which was accounts for 33.3%. In none of these patients was the coon affected. There was no retention or other side effects observed in this study.

**Table 2:** The characteristics of VCE at small intestinal in pediatric HSP patients

VCE findings	Number (Percentage)
Edema/Congestion	12 (41.4%)
Mucosal erythema or petechia	20 (69.0%)
Mucosal erosion	23 (79.3%)
Multiple ulcers	17 (58.6%)

*VCE* Video capsule endoscopy; *HSP* Henoch–Schönlein purpura

**Table 3:** The disease location detected by video capsule endoscopy

Disease location	Number (Percentage)
Esophagus	0 (0.0%)
Stomach	3 (10.0%)
Duodenum bulb	6 (20.0%)
Descent of duodenum	10 (33.3%)
Jejunum	29 (96.7%)
Ileum	13 (44.3%)
Total	30

The EGD, VCE, and colonoscopy findings of HSP patients with or without skin purpura were shown in Table 4.

**Table 4:** Number of patients detected typical lesions of HSP in patients with or without skin purpura by EGD, colonoscopy and VCE

	With skin purpura *N* = 13	Without skin purpura *N* = 17	Total
EGD	8	4	12
Colonoscopy	0^a^	2	2
VCE	12^b^	17	29

*HSP* Henoch–Schönlein purpura; *EGD* Esophagogastroduodenoscopy; *VCE* Video capsule endoscpy

a. Colonoscopy detected polyp by colonoscopy in one patient was not considered related to HSP; b. VCE did not pass the gastric pylorus in one patient

### Treatment

Ninety percent (27/30) of the patients initially treated with corticosteroids and another three patients received proton pump inhibitors or montelukast for mild gastrointestinal symptoms. Four patients were treated by immunoglobin combined with steroids, and seven patients were treated by immunosuppressants, as they were not completely responsive to steroids or dependent on steroids. One patient with intestinal perfusion had surgery and then treated with methotrexate for two months. All the patients were followed in our center and completely recovered.

## Discussion

VCE was approved to be used in children over two years of age by the US FDA in 2009 [3, 4]. The most frequent indications for VCE in children are inflammatory bowel disease (IBD), obscure gastrointestinal bleeding (OGIB), malabsorption, protein-losing enteropathies, abdominal pain, small bowel polyps and tumors [5].

HSP is a systemic small-vessel vasculitis diagnosed mainly based on the clinical manifestation and pathological study of the purpura. Diagnosis of HSP usually does not need endoscopy examination. However, in some HSP patients both EGD and VCE may be necessary. And most of the HSP patients with GI symptoms did undergo an EGD procedure which revealed the typical mucosal features of HSP in the proximal small bowel. The minority of these HSP patients who had a negative EGD finding were asked to undergo the VCE procedure. The VCE results in these children demonstrated extensive HSP involvement in the jejunum and rest of the small bowel similar to the findings in the duodenum. Thus, the possible HSP patients who have minimal or no HSP rash, have some abdominal symptoms and signs, and have an unremarkable EGD finding would likely benefit from a VCE to confirm or eliminate the possibility of the HSP diagnosis. The VCE may also indicate the extent and severity of the small-vessel vasculitis GI disease in any child and affect treatment decisions.

To our best knowledge, there is no study focused on the VCE examination of pediatric HSP patients. The typical findings detected by VCE in the small intestinal is similar to the EGD findings in other adult cohort report [6], presenting with mucosal edema, congestion, erosion, sporadic purpura or diffuse purpura, and usually with multiple irregular superficial ulcers. In our cohort, the most frequently involved part of the GI tract was the jejunum. All the children had their jejunum involved excluding the child whose capsule did not pass the pylorus. It was different from the reported literature by Eon Jeong Nam et al. [2]. In their report with a series of adult HSP patients, the second part and the terminal ileum were the most frequently involved parts of HSP patients, and the colon was frequently involved as well. However, the VCE or small intestinal endoscopy was not performed in this study; thus, the jejunum was not assessed. On the contrary, in our cohort, none of the patients had the colon involved. The difference between the studies could partially be explained by the age of patients in the study. The mean age of patients was 96.7 months in our cohort while the patients were adults in the study of Eon Jeong Nam et al.. And also, the location and extent of disease depend on the time of performing endoscopy and the severity of disease. The median time of our patients receiving VCE was around three weeks.

It is accessible to diagnosis HSP with typical symptoms and skin purpura according to the diagnostic criteria [7]. HSP is reported to share similar clinical manifestation and sometimes has a colonoscopy appearance that resembles ulcerative colitis [8]. The use of VCE in these patients may help delineate the diagnosis, document the extent and severity of the vasculitis, and aid in choosing the best treatment. It needs further study whether patients with extensive small bowel involved and with more severe mucosal lesions need longer duration of steroid treatment. Although HSP is self-limited, we observed that patients with more severe HSP small intestinal inflammation by VCE often suffered a longer disease course, even as long as 8 months.

MRE and CT were also performed in 14 patients, and 71.4% of them detected thickening of the small intestinal wall. Compared with VCE, the MRE and CT finding of these patients are not typical, and could not identify superficial ulcers of mucosa or evaluate the condition of bleeding.

This study has some limitations. First, this was a retrospective study with a small cohort of patients. The study only included patients diagnosed by VCE as HSP, not all the HSP patients with GI symptoms were evaluated. Second, all of the patients had EGD and VCE in this study. But not all patients had a colonoscopy examination. Thus, the lesions in the colon may be omitted in some patients. Third, some patients could not be definitely diagnosed to have HSP in our estimation due to limited GI symptoms and signs and VCE findings. However, these patients were all followed up in our center, and the outcome of these patients were good.

## Conclusions

In conclusion, this is the first cohort study focused on the VCE findings in pediatric HSP patients. The typical discovery of endoscopy could make the diagnosis of HSP with or without palpable purpura. VCE is a safe and effective study technique to diagnosis suspected but not confirmed HSP. In other words, VCE may be the best modality to better evaluate children suspected to have HSP but who do not have the typical HSP petechiae and purpura present or a helpful EGD. The VCE can evaluate parts of the small intestine the EGD cannot and that may make a tremendous difference in these patients.

## Data Availability

All data generated or analysed during this study are included in this published article.

## Abbreviations

HSP: Henoch–schönlein purpura
GI: Gastrointestinal
EGD: Esophagogastroduodenoscopy
VCE: Video capsule endoscopy
IQR: Interquartile range
MRE: Magnetic resonance enterography
CT: Computed tomography
IBD: Inflammatory bowel disease
OGIB: Obscure gastrointestinal bleeding
