# Correction: the characteristics of video capsule endoscopy in pediatric Henoch–Schönlein purpura with gastrointestinal symptoms

**DOI:** 10.1186/s12969-022-00683-w

**Published:** 2022-05-02

**Authors:** Youhong Fang, Kerong Peng, Hong Zhao, Jie Chen

**Affiliations:** Department of gastroenterology, The Children’s Hospital, Zhejiang University School of Medicine, National Clinical Research Center for Child Health, 3333 Bin Sheng Road, Hangzhou, 310052 Zhejiang Province China

**Correction to: Pediatr Rheumatol 18, 84 (2020)**

**https://doi.org/10.1186/s12969-020-00471-4**

The original publication of this article contained 2 errors:

1. An incorrect funding number was used
2. The time for patient enrolment was incorrect in 2 locations

The incorrect and correct information is shown in this correction article. The original article has been updated.

**Incorrect**

- This research is supported by the Natural Science Foundation of Zhejiang Province, China (**LQ19H030005**). The funder had no role in study design, data collection and analysis, decision to publish, or preparation of the manuscript.
- Patients diagnosed with HSP and analyzed by VCE examination at our hospital from **February 2010 to January 2019** are enrolled.
- We enrolled 30 HSP patients who had a VCE examination at Children’s Hospital, Zhejiang University School of Medicine from **February 2010 to January 2019.**

**Correct**

- This research is supported by the Natural Science Foundation of Zhejiang Province, China (**LQ19H030010**). The funder had no role in study design, data collection and analysis, decision to publish, or preparation of the manuscript.
- Patients diagnosed with HSP and analyzed by VCE examination at our hospital from **February 2010 to January 2020** are enrolled.
- We enrolled 30 HSP patients who had a VCE examination at Children’s Hospital, Zhejiang University School of Medicine from **February 2010 to January 2020.**

